# Structural and Mechanistic Perspectives on SARS-CoV-2 Nonstructural Protein 14-Mediated Cap Formation and Drug Discovery

**DOI:** 10.3390/microorganisms14081815

**Published:** 2026-08-18

**Authors:** Yifan Zhao, Rhea Guo, Yang Yang, Chang Liu

**Affiliations:** 1 Department of Biochemistry and Molecular Biology, Bloomberg School of Public Health, Johns Hopkins University, Baltimore, MD 21205, USA; 2 Brain and Cognitive Sciences Department, School of Arts and Sciences, University of Rochester, Rochester, NY 14627, USA; 3 Computer Science Department, Hajim School of Engineering and Applied Sciences, University of Rochester, Rochester, NY 14627, USA; 4 Roy J. Carver Department of Biochemistry, Biophysics and Molecular Biology, Iowa State University, Ames, IA 50011, USA

**Keywords:** SARS-CoV-2, coronavirus, nsp14, methyltransferase, RNA capping, antiviral development

## Abstract

SARS-CoV-2 relies on a virus-encoded RNA capping pathway to produce 5′ cap structures that are essential for mRNA stability, efficient translation, and evasion of host innate immune surveillance. Within this pathway, nonstructural protein 14 (nsp14) catalyzes N7 methylation of the guanine cap, a key step that converts the cap core into a functional Cap-0 structure and enables subsequent maturation. Owing to its essential role in viral replication and its high conservation across coronaviruses, nsp14 has emerged as an attractive antiviral target. Recent structural and biochemical studies have elucidated the architecture of the nsp14 N7-methyltransferase domain, revealing an S-adenosyl-L-methionine (SAM)-dependent fold with a defined cofactor-binding site and an adjacent cap-binding pocket that orients the RNA substrate for methyl transfer. These insights have guided the development of diverse inhibitor classes, including SAM-competitive analogs, bisubstrate-like compounds, and non-nucleoside inhibitors identified through screening approaches. While early SAM-like inhibitors demonstrated target tractability, their therapeutic potential has been limited by challenges in selectivity and cellular permeability. More recent inhibitors that target the cap-binding pocket or exploit product-assisted ternary complex mechanisms highlight alternative strategies for achieving improved potency and specificity. Despite these advances, current structural models rely on truncated RNA substrates and isolated protein constructs, which may not fully capture the native catalytic environment. Future efforts to resolve nsp14 within the replication–transcription complex and develop novel inhibition strategies will be critical for advancing mechanistic understanding and antiviral development.

## 1. Introduction

Although the acute phase of the COVID-19 pandemic has subsided, severe acute respiratory syndrome coronavirus 2 (SARS-CoV-2), officially classified as *Betacoronavirus pandemicum* within the subgenus *Sarbecovirus*, the causative agent of COVID-19, continues to circulate globally in 2025, contributing to persistent morbidity, viral evolution, and ongoing public health burden [1]. The emergence of immune-evasive variants and the continued need for antiviral preparedness underscore the importance of understanding conserved viral enzymatic mechanisms that can serve as a robust therapeutic target [2].

**Figure 1:**
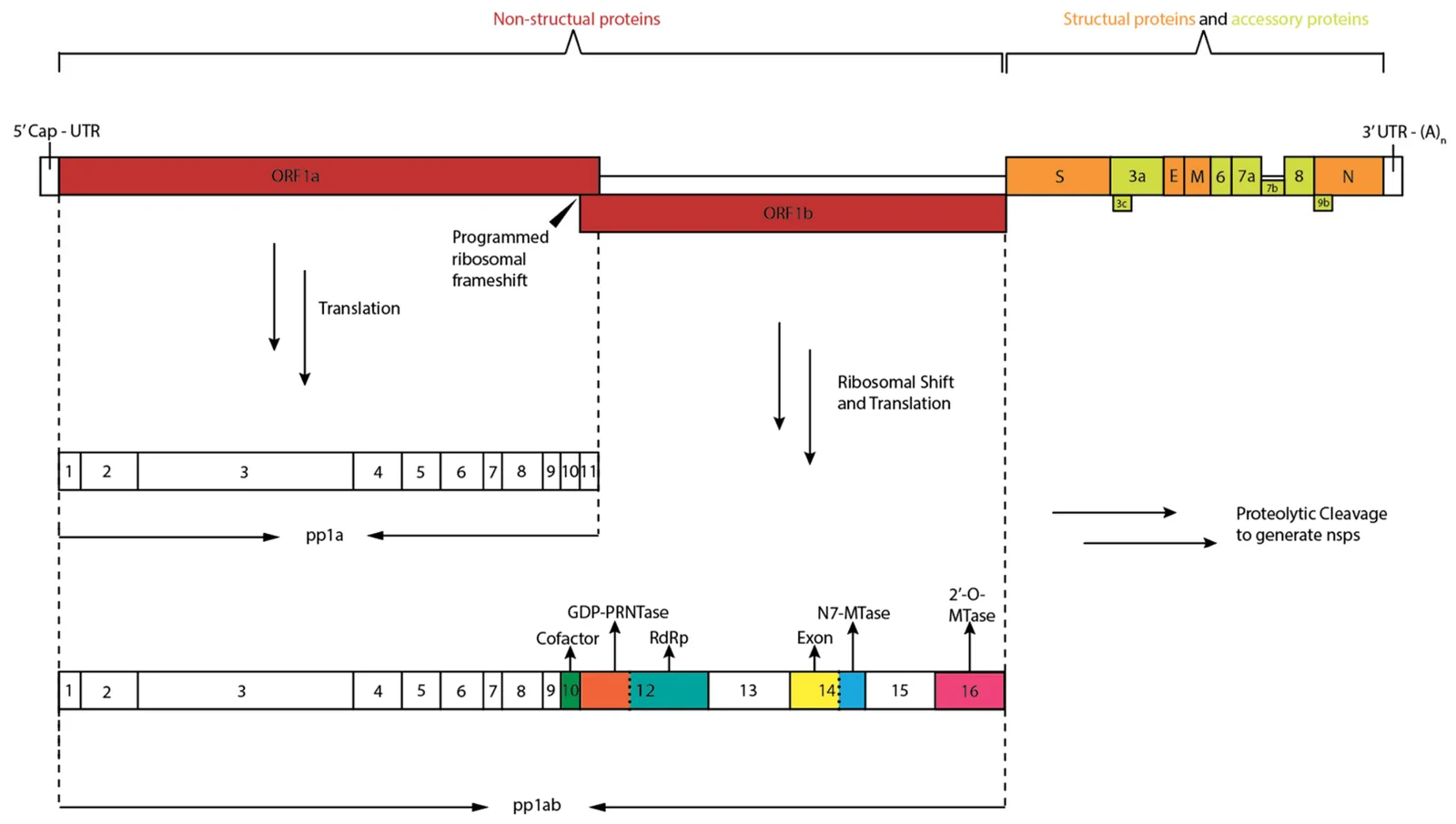
Schematic representation of the SARS-CoV-2 genome organization. ORF1a and ORF1b encode the viral nonstructural proteins (nsp1–16), which are generated through translation of the pp1a and pp1ab polyproteins followed by proteolytic processing. The downstream region encodes the structural proteins spike (S), envelope (E), membrane (M), nucleocapsid (N), and multiple accessory proteins. The nonstructural proteins involved in viral RNA cap synthesis are highlighted in different colors.

SARS-CoV-2 is an enveloped, positive-sense single-stranded RNA virus belonging to the genus Betacoronavirus [3]. Its ~30 kb genome is among the largest of RNA viruses and shares approximately 82% sequence identity with the severe acute respiratory syndrome coronavirus (SARS-CoV) genome [4,5]. The genome is organized into a 5′ untranslated region (UTR), two large open reading frames (ORF1a and ORF1ab), multiple downstream structural and accessory genes, and a 3′ UTR. The structural proteins mediate viral entry, assembly, and genome packaging. In addition, accessory proteins regulate host pathways such as interferon signaling and apoptosis, thereby promoting immune evasion and viral fitness [6,7,8,9,10,11,12]. ORF1a and ORF1ab are translated into the polyproteins pp1a and pp1ab, which are proteolytically processed into 16 nonstructural proteins (nsps) (Figure 1). Part of the nsps assemble into the replication–transcription complex (RTC), a dynamic multi-protein machinery responsible for viral RNA synthesis, proofreading, RNA modification, and transcript maturation [13]. The central catalytic subunit of the RTC is nsp12, the RNA-dependent RNA polymerase (RdRp), which cooperates with accessory factors such as nsp7 and nsp8 to coordinate genome replication and subgenomic mRNA production [14,15].

A critical step in viral RNA maturation is the formation of a 5′ cap structure. RNA capping ensures efficient translation by host ribosomes, protects viral transcripts from 5′→3′ exonucleolytic degradation, and enables evasion of innate immune sensors that detect uncapped or improperly capped RNA species [16].

In SARS-CoV-2, the viral capping pathway involves sequential enzymatic steps coordinated by multiple nsps. Following the initial GDP polyribonucleotidyltransferase (PRNTase) reaction by the Nidovirus RdRp-associated nucleotidyl transferase (NiRAN) domain of nsp12 and subsequent transfer reactions involving nsp9 [17,18,19,20,21], the C-terminal N7-methyltransferase (N7-MTase) domain of nsp14 catalyzes the critical first methylation step. Specifically, nsp14 N7-MTase methylates the guanine residue at the N7 position to form the Cap-0 structure [22]. This N7-methylation is subsequently followed by nsp16-catalyzed 2′-O-methylation of the first transcribed nucleotide to generate the Cap-1 structure [23,24,25,26]. Within this viral cascade, the nsp14 N7-MTase activity is therefore a key determinant of viral cap maturation and host immune evasion. Genetic and biochemical studies have demonstrated that disruption of coronavirus N7-MTase activity leads to severely attenuated viral replication, impaired viral RNA translation, and enhanced sensitivity to innate immune restriction [27,28]. In related coronaviruses, mutation of conserved residues within the N7-MTase active site abolishes enzymatic activity and yields a nonviable or replication-defective virus [29]. Moreover, incomplete cap methylation renders viral RNA more susceptible to interferon-mediated restriction and recognition by host sensors [30]. These findings establish N7 methylation not merely as a structural modification but as a functional checkpoint required for productive infection.

N7 methylation of the 5’ cap is a universal feature of eukaryotic mRNA and is equally essential for coronavirus replication. However, because coronaviruses encode their own N7-methyltransferase (nsp14) that is structurally distinct from host capping machinery, this enzyme has emerged as a compelling target for selective antiviral intervention [31,32]. A detailed understanding of its structural organization, catalytic mechanism, and ligand recognition principles is therefore critical. In the sections that follow, this review examines the structural framework and mechanistic basis of SARS-CoV-2 N7-MTase function and reevaluates current efforts to exploit this enzyme for therapeutic intervention.

## 2. Molecular Mechanism of RNA Cap N7 Methylation by nsp14

**Figure 2:**
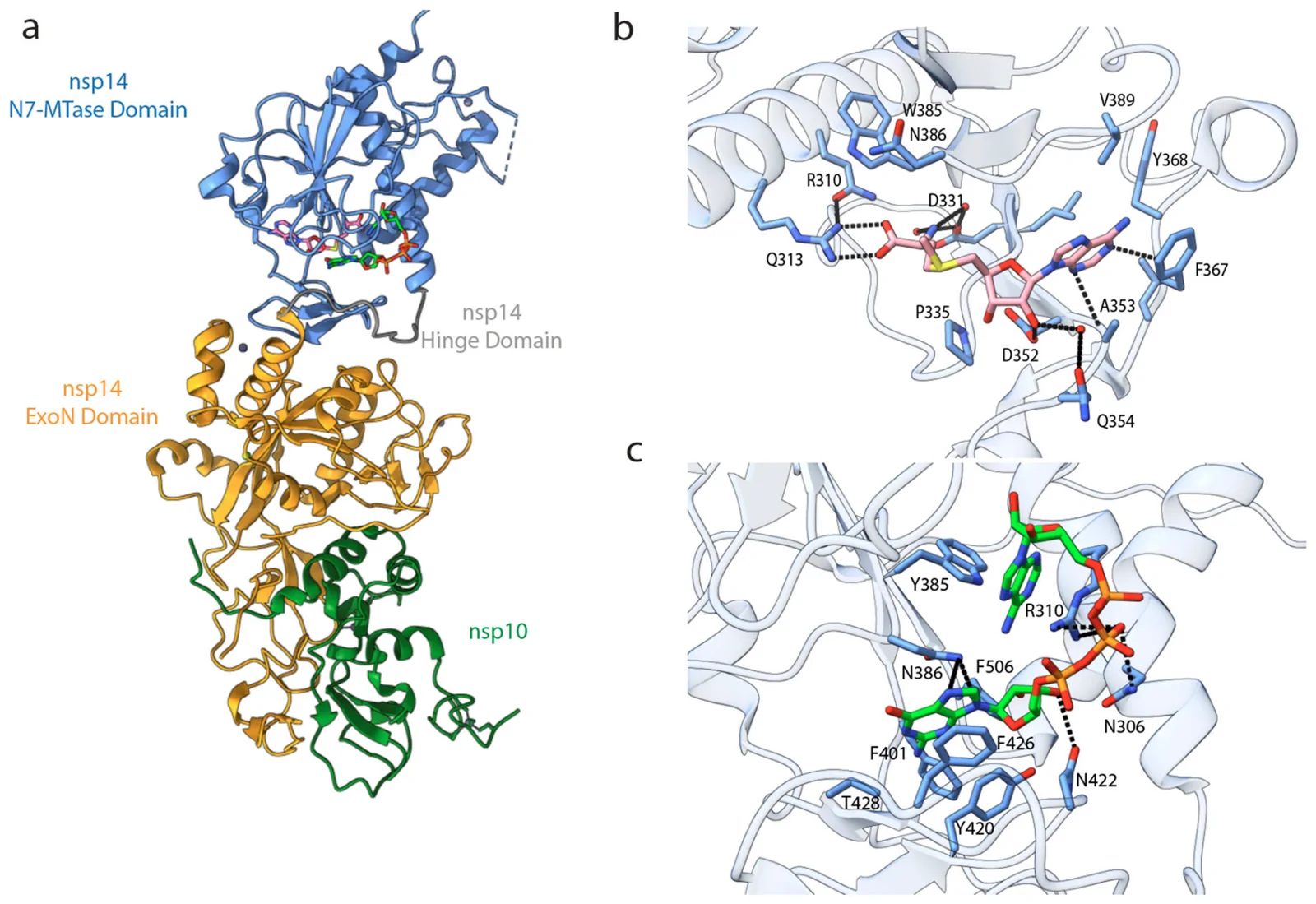
Structures of SARS-CoV-2 and SARS-CoV nsp14 in complex with SAH and GpppA: (**a**) Overall architecture of SARS-CoV nsp14-SAH-GpppA complex (PDB: 5C8S), highlighting the N7-MTase domain (blue), ExoN domain (yellow), and hinge region (gray). The nsp10 (forest green) interacts with the nsp14 ExoN domain. (**b**) SAM-binding site of SARS-CoV-2 nsp14 (PDB: 7TW7), with key residues shown as sticks (cornflower blue) and SAM in pink. (**c**) GpppA-binding site of SARS-CoV nsp14 (PDB: 5C8S), with key residues in stick representation (cornflower blue) and GpppA in lime. Key interactions are indicated by dashed lines. Because no high-resolution SARS-CoV-2 structure containing a canonical Cap-0 RNA substrate is currently available, the cap-binding interactions shown in panel C represent a structural model inferred from SARS-CoV and extrapolated to SARS-CoV-2 based on the high conservation of the active site.

SARS-CoV-2 nsp14 is a bifunctional protein comprising an N-terminal 3′-to-5′ exoribonuclease (ExoN) domain and a C-terminal S-adenosyl-L-methionine (SAM)-dependent N7-MTase domain [33,34]. The nsp14 N7-MTase domain catalyzes guanine N7-methylation of the cap core, converting it to the Cap-0 structure (m7GpppA). While the ExoN activity requires association with the viral cofactor nsp10, the N7-MTase domain does not (Figure 2a). The N7-MTase domain adopts an unusual SAM-dependent methyltransferase fold. Structurally, it consists of a five-stranded β-sheet flanked by surrounding α-helices, a topology that differs from the central seven-stranded β-sheet architecture observed in many Class I methyltransferases [35]. The SAM-binding pocket is embedded within this conserved scaffold, where the adenine base of SAM is accommodated in a hydrophobic cavity and stabilized by a network of hydrogen bonds and solvent-mediated interactions [36]. Adjacent to the cofactor-binding site lies the cap-binding groove, which positions the guanine base of the RNA substrate in close proximity to the reactive methyl group of SAM. The spatial arrangement of these two pockets enforces strict geometric constraints that are essential for catalytic precision [37]. Mechanistically, nsp14 catalyzes the transfer of a methyl group from SAM to the N7 position of the guanine cap via an in-line S_N_2-type reaction. High-resolution structural analyses of SARS-CoV-2 nsp14 reveal that the N7-MTase domain is connected to the N-terminal ExoN domain through a flexible hinge region, suggesting interdomain communication that modulates enzymatic activity [38]. Excluding this hinge, the N7-MTase cores of SARS-CoV and SARS-CoV-2 are nearly identical, underscoring strong structural conservation.

In an nsp14 N7-MTase-SAM/SAH (S-adenosyl-L-homocysteine)/Sinefungin co-crystal structure, the adenine moiety is nestled within a predominantly hydrophobic cavity defined by Phe367, Tyr368, Val389, and Ala353, while its polar atoms are stabilized by hydrogen bonds involving the backbone of Tyr368 and Ala353 (Figure 2b). The ribose group is anchored by a direct interaction with the conserved residue Asp352 and additional solvent-mediated contacts with Gln354, consistent with its essential role in catalysis. The homocysteine tail of the ligand is positioned by a network of interactions involving Arg310, Gln313, Gly333, Asp331, Trp385, and Asn386, many of which are bridged by ordered water molecules that contribute substantially to binding [39]. Binding studies show that SAH associates with nsp14 with substantially higher affinity than SAM or Sinefungin, likely due to favorable water-mediated interactions near Asn386, highlighting SAH as a particularly attractive scaffold for inhibitor development. Although the MTase domain alone is catalytically competent, its activity and ligand-binding affinity are reduced in the absence of the ExoN domain, implying a stabilizing allosteric effect between the two domains [40].

To date, a high-resolution structure of the SARS-CoV-2 nsp14 N7-MTase in complex with a canonical Cap-0 RNA substrate has not been reported. The structure model that shows interactions with the RNA substrate is therefore inferred from the homologous SARS-CoV nsp14-GpppA complex. This extrapolation is supported by the high degree of conservation between the two enzymes: the SARS-CoV-2 N7-MTase domain shares approximately 98% sequence conservation with its SARS-CoV counterpart, while the SAM/SAH-binding pocket and GpppA-binding pocket exhibit 100% and 98% conservation, respectively [41,42,43]. Unless otherwise stated, the following discussion of cap recognition refers to this SARS-CoV-derived structural model and is extrapolated to SARS-CoV-2 on the basis of this conservation. In this model, the structure of the RNA substrate is the guanine cap and the first transcribed nucleotide, specifically GpppA (Figure 2c). The cap analog binds adjacent to the SAM-binding site within a tightly confined pocket of the nsp14 N7-MTase. The guanine base is stabilized by a cluster of aromatic and hydrophobic residues, among which Phe426 plays a dominant role, as its mutation leads to a substantial loss of enzymatic activity. Asn386, positioned near the methyl transfer site, forms critical hydrogen bonds with the guanine moiety and is essential for proper substrate orientation during catalysis. Additional contacts with the ribose and triphosphate groups, mediated by conserved polar and basic residues such as Asn306, Arg310, and Lys336, contribute to substrate binding and catalytic efficiency, with Arg310 being particularly important [43]. Stacking interactions involving Trp385 further stabilize the cap structure. Collectively, these interactions constrain both SAM and GpppA within a highly restricted pocket, enforcing precise geometric and electrostatic complementarity that positions the SAM methyl group in close proximity to the N7 atom of guanine, thereby enabling efficient in-line methyl transfer [42].

It is important to note that the RNA substrates employed in these structural studies are primarily limited to the guanine cap and the first transcribed nucleotide, specifically GpppA. Consequently, the interactions observed between this truncated substrate and nsp14 may diverge from those occurring with actual nascent RNA. Obtaining structural data using longer capped RNA sequences remains essential for establishing a more biologically relevant model of the nsp14-RNA-SAH complex in the future. Despite these limitations, the available SARS-CoV and SARS-CoV-2 structures have significantly advanced our understanding of the specific residues critical for stabilizing the transition state and executing the catalytic reaction.

## 3. Development of Versatile nsp14 Inhibitors

Most clinically used antivirals against SARS-CoV-2 target either viral protease or RdRp. For example, the protease inhibitor nirmatrelvir (used in combination therapy) targets the main protease (Mpro), while nucleoside analogs such as remdesivir and molnupiravir inhibit the viral RdRp. Although these strategies have demonstrated clinical efficacy, they remain susceptible to resistance. Moreover, they primarily target viral proteolysis and RNA replication, leaving other essential processes comparatively underexplored for antiviral intervention [44,45,46,47]. Viral RNA cap synthesis represents another essential step in the viral life cycle that has received comparatively less attention as a target for therapeutic intervention. The structural features of nsp14 differ substantially from those of the host cap N7-methyltransferase RNMT, including limited sequence conservation within the active site and a more restricted binding pocket. These differences provide opportunities for selective inhibitor design, although achieving selectivity over host methyltransferases remains a major challenge [48,49].

Several complementary approaches have been employed to identify nsp14 inhibitors, including structure-guided design, virtual screening, high-throughput biochemical screening, and drug repurposing. These efforts have yielded multiple mechanistically different classes of inhibitors, including SAM-competitive inhibitors, bisubstrate inhibitors, and non-SAM inhibitors (Table 1). In a structure-based study, Otava et al. reported the design of nanomolar nsp14 inhibitor compound 16 (TO507) using a homology model derived from the closely related SARS-CoV nsp14 enzyme [50]. The authors identified a lateral hydrophobic cavity adjacent to the SAM-binding pocket, formed by residues including Val287, Arg289, Phe367, Val389, and Pro429 [50]. Guided by this observation, the authors designed 7-deaza-SAH analogs bearing aromatic groups attached through optimized linkers to extend into the lateral cavity. Extension of aromatic substituents into this cavity yielded inhibitors with low-nanomolar biochemical potency and strong binding affinity. Among all these inhibitors, compound 16 demonstrated favorable selectivity toward the viral N7-methyltransferase, inhibiting SARS-CoV-2 nsp14 with an IC_50_ of 3 nM while exhibiting an approximately 170-fold weaker activity against hRNMT (IC_50_ = 500 nM). However, selectivity across the broader methyltransferase family remained limited, as the compound retained potent activity against several host methyltransferases, including protein arginine methyltransferase 7 (PRMT&) and DNA methyltransferases (DNMTs), highlighting the continued challenge of achieving broad host methyltransferase selectivity [50]. Also, the zwitterionic nature of the compounds limited cellular permeability and antiviral activity.

In another study, Devkota et al. reported several SAH-derived compounds with measurable inhibitory activity, including SS148, a nitrile-substituted SAH analog that inhibited nsp14 with an IC_50_ of ~70 nM in biochemical assays. Kinetic analyses indicated that SS148 acts as a SAM-competitive inhibitor, consistent with binding to the canonical cofactor-binding pocket of the methyltransferase [41]. In the same study, the compound DS0464 displayed weaker biochemical potency (IC_50_ ≈ 1.1 µM) but exhibited an alternative inhibition profile. Kinetic experiments suggested that DS0464 competes with both the SAM cofactor and the RNA substrate, a behavior consistent with a bisubstrate-like mode of inhibition. Docking models proposed that DS0464 occupies the SAM-binding site while extending toward the RNA cap-binding region, although this binding mode has not been confirmed structurally. This different binding mode may have to do with the broader selectivity range of DS0464. While SS148 was selective against all 20 SET domain lysine MTases tested in the paper, it still inhibited multiple class I human methyltransferases, including RNA, DNA, and arginine methyltransferases. Compared with SS148, DS0464 exhibited improved selectivity, inhibiting only 5 of 33 human methyltransferases tested, suggesting that simultaneously engaging the cofactor- and substrate-binding regions may improve target selectivity [41]. However, neither SS148 nor DS0464 demonstrated antiviral activity in cell-based assays [41].

Progress has also been made in bi-substrate inhibitor development. Ahmed-Belkacem et al. reported the synthesis of a series of adenine dinucleosides designed to mimic features of the methyl transfer transition state [51]. Although initially conceived as inhibitors of 2′-O-methyltransferases, several compounds unexpectedly inhibited the N7-MTase activity of SARS-CoV nsp14. Among these, a nitro-substituted arylsulfonamide-linked dinucleoside (compound 13) exhibited submicromolar biochemical potency (IC_50_ ≈ 0.6 μM) and showed substantially reduced inhibition of the human RNA N7-MTase compared with sinefungin [51]. Kottur et al. reported crystal structures of the nsp14 MTase core in complex with SGC0946 and SGC8158, two inhibitors originally developed against host methyltransferases [52]. Structural and calorimetric analyses indicated that these compounds occupy the SAM-binding site while also disrupting the binding of a GpppA cap analog, suggesting that inhibition involves perturbation of the cap-binding region in addition to cofactor competition. Notably, both inhibitors were evaluated in cell-based infection assays, where SGC0946 and a prodrug form of SGC8158 reduced viral replication in HeLa-ACE2 cells [52].

Non-nucleotide inhibitors are mainly derived from various screenings. A study explored whether previously characterized bioactive compounds could have an inhibitory effect. From this effort, several molecules with distinct pharmacological origins were identified as nsp14 inhibitors in vitro [53]. These included lomeguatrib, a potent inhibitor of the human O’6-methylguanine-DNA methyltransferase (MGMT) originally developed for oncology applications [54]; PF-03882845, a mineralocorticoid receptor agonist investigated in metabolic disease [55]; trifluperidol, an antipsychotic drug with known dopaminergic activity [56]; and inauhzin, a small molecule reported to modulate Sirtuin 1 (SIRT1) and p53 signaling pathways [57]. Despite their diverse mechanisms and lack of prior association with viral enzymes, several of these compounds reduced SARS-CoV-2 infection in Vero E6 cells. Through large-scale similarity and substructure searches across ultra-large chemical libraries, conservative analogs of multiple non-nucleoside hit series were identified, prioritized by predicted binding within the SAM-binding pocket, and evaluated experimentally. Modest improvements in biochemical potency were achieved across several chemotypes. In particular, analogs derived from ZINC475239213 and ZINC730084824 retained or modestly enhanced inhibitory activity following substituent refinement, while fragment-based non-nucleoside hits such as ZINC61142882 were successfully elaborated to yield inhibitors with IC_50_ values below 10 µM. Among the analogs derived from the docking hit ZINC61142882, the optimized compound Z795161988 retained low-micromolar biochemical potency against nsp14 (IC_50_ = 2.2 μM in the enzymatic assay during lead optimization; 6 μM in the selectivity assay) and exhibited an SAM- and RNA-competitive inhibition pattern. However, profiling against a panel of 30 human methyltransferases revealed only modest selectivity, with measurable inhibition of nine host enzymes (IC_50_ = 4–26 μM). Moreover, evaluation in cell-based antiviral assays did not demonstrate a clear antiviral effect independent of cytotoxicity, indicating that additional medicinal chemistry efforts will be required to improve both target selectivity and cellular safety [58].

**Figure 3:**
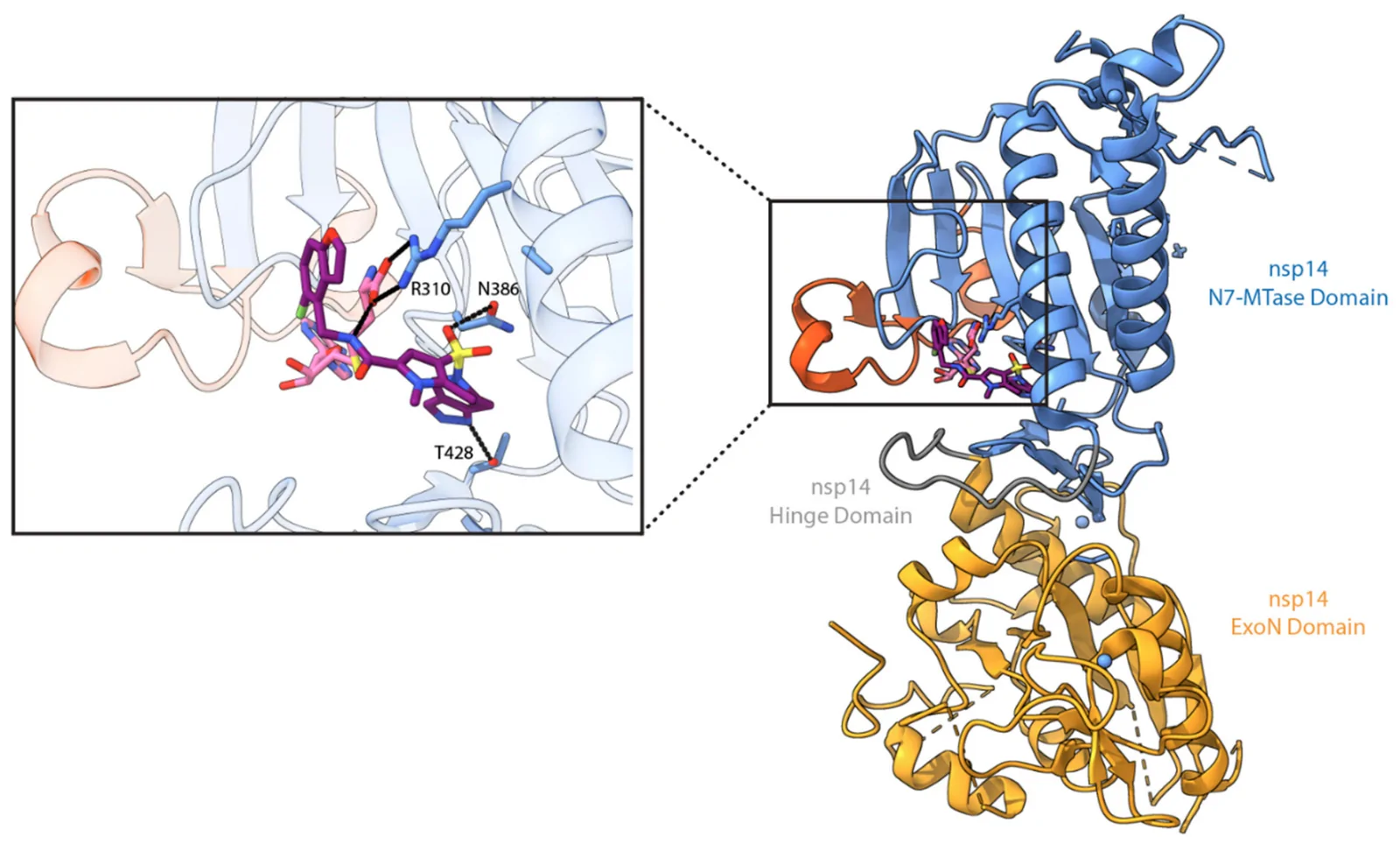
Crystal structure of TDI-015051 bound to SARS-CoV-2 nsp14 in complex with the cofactor SAH (PDB: 8R7B). TDI-015051 (purple) binds to the nsp14 N7-MTase domain (blue) in the presence of SAH (pink), resulting in the formation of a ternary complex. The Asp352–Phe377 “lid” region (red) undergoes a conformational rearrangement that encloses both SAH and the inhibitor within the active site, thereby stabilizing the ternary complex and contributing to product-assisted inhibition.

More recently, a large-scale biochemical screening campaign targeting full-length SARS-CoV-2 nsp14 identified a non-SAM-competitive inhibitor that, following structure-guided optimization, yielded the lead compound TDI-015051, exhibiting picomolar biochemical potency and antiviral activity in cell-based assays [59]. Unlike classical SAM-competitive methyltransferase inhibitors, TDI-015051 operates through a product-assisted ternary complex mechanism, which represents a relatively rare and underexplored mode of inhibition [60]. The compound preferentially binds the nsp14-SAH complex, forming a stable nsp14-SAH-inhibitor assembly characterized by slow dissociation kinetics. Structural analyses revealed that the inhibitor occupies the guanine cap-binding pocket rather than the SAM site, sterically blocking GpppA-containing RNA substrates. Binding is further stabilized by a conformational “lid” rearrangement in the Asp352-Phe377 region that encloses SAH and the inhibitor within the active site (Figure 3). This mechanism differs from previously reported SAM-competitive strategies by exploiting binding to the enzyme-product complex rather than directly competing with the methyl donor. In cellular systems, TDI-015051 suppressed SARS-CoV-2 replication, consistent with impaired cap formation and reduced viral RNA translation. Notably, increasing intracellular SAH levels enhanced antiviral potency, supporting the proposed ternary-complex mechanism in cells. The compound demonstrated selectivity, with no detectable inhibition of host RNMT in complex with its activating cofactor RAM (RNMT Activating Miniprotein) or the viral nsp16/nsp10 2′-O-methyltransferase, and resistance mutations clustered near the cap-binding pocket imposed significant viral fitness costs. In a K18-hACE2 mouse model, oral administration reduced lung viral titers with efficacy comparable to nirmatrelvir and showed synergistic effects in combination therapy [59]. Collectively, these findings establish TDI-015051 as the most extensively characterized non-SAM-competitive nsp14 inhibitor reported to date and provide proof of concept for product-assisted inhibition of the viral N7-methyltransferase.

## 4. Future Perspectives and Conclusions

The identification of SARS-CoV-2 nsp14 as a druggable enzymatic target has important implications that extend beyond understanding viral RNA capping. Unlike spike-directed interventions, which are vulnerable to antigenic drift, the N7-MTase operates within a highly conserved and functionally constrained pathway of viral RNA maturation [61]. This makes nsp14 an attractive target not only for current SARS-CoV-2 variants but also for future emerging coronaviruses. Studies have demonstrated that some nsp14 inhibitors retain activity against multiple coronaviruses, supporting the concept that this conserved enzyme may be amenable to broader antiviral targeting. Nevertheless, this area of research is still in its early stages, and further medicinal chemistry optimization, together with evaluation across a broader range of coronaviruses, will be required to determine the extent of their antiviral spectrum.

Nsp14 does not function in isolation. It operates within a large, dynamic RTC that integrates RNA synthesis, proofreading, and cap maturation. Although individual components of this machinery have been structurally characterized and integrative models of the RTC have been proposed, whether these models are physiologically relevant remains to be validated [15,62]. Placing nsp14 within its native multiprotein context will be essential for understanding how methylation is coordinated with polymerase progression, whether conformational coupling modulates enzymatic activity, and how protein–protein interactions influence catalytic efficiency. Accordingly, reconstituting the architecture of an RTC that includes nsp14 in an actively transcribing state is likely to be a key objective for future studies.

Despite rapid progress in defining the structural framework of the SARS-CoV-2 nsp14 N7-MTase, the field remains constrained by models that only partially recapitulate the natural catalytic environment. Most available structures rely on minimal cap analogs, which capture core interactions but do not reflect the steric, electrostatic, and conformational context of extended viral RNA. Given that N7 methylation occurs co-transcriptionally within the RTC, future efforts must incorporate longer capped RNA substrates and aim to reconstruct nsp14 within a larger, dynamic RTC assembly. Such systems will be critical for achieving a physiologically relevant understanding of cap maturation during viral replication.

From a therapeutic perspective, inhibitor development has clearly demonstrated that the N7-MTase pocket is chemically tractable. However, many SAM-like inhibitors suffer from limited cellular permeability, suboptimal pharmacokinetics, or insufficient antiviral potency despite strong biochemical activity. Further medicinal chemistry efforts will need to move beyond simple cofactor mimicry to improve drug-like properties while preserving selectivity over host methyltransferases. Strategies that target adjacent pockets, exploit cap-binding interactions, or stabilize specific conformational states of nsp14 may offer more favorable translational potential. Additionally, targeting protein–protein interfaces, such as potential interaction surfaces between nsp14 and its regulatory partners, including nsp10, may provide alternative avenues for modulating enzymatic activity in a context-dependent manner. Similar strategies have been applied to the nsp16–nsp10 complex, where disruption of their interaction impairs 2′-O-methyltransferase activity and may allosterically affect SAM and RNA binding [63,64]. Ultimately, advancing our understanding of nsp14 will require integration of structural biology, enzymology, and cell-based validation within a more physiologically relevant framework. Achieving these goals will not only refine our understanding of coronavirus RNA cap maturation but also strengthen the foundation for the development of durable antiviral strategies targeting conserved enzymatic processes.

## Figures and Tables

**Table 1:** Structural and biochemical/cellular profiles of nsp14 inhibitors.

**Name**	**Structure**	**Type**	**IC_50_/EC_50_ Value**
Compound 16	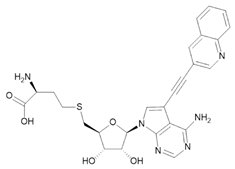	SAH-derived Inhibitor	IC_50_ = 3 nM
Compound 13	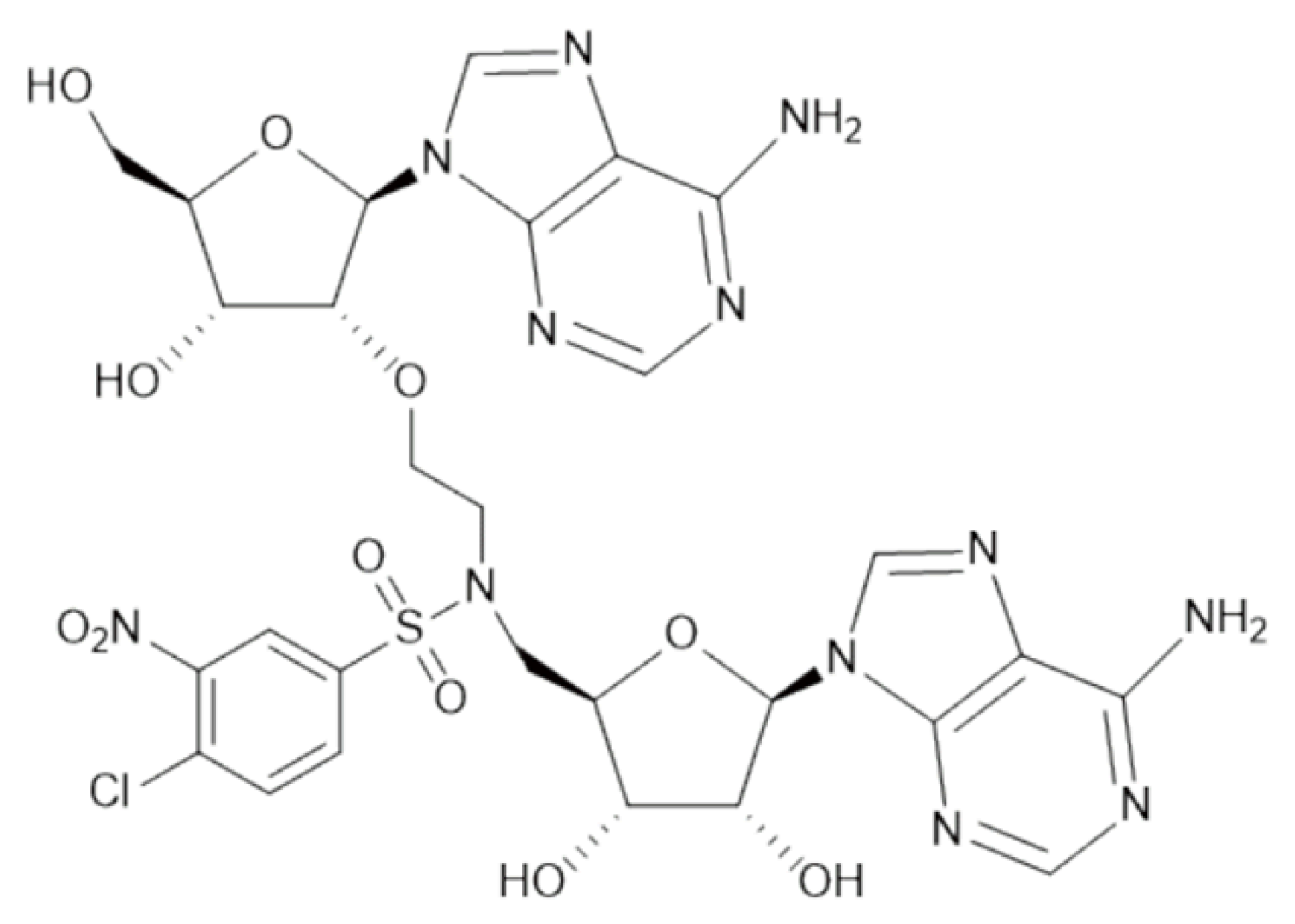	Bisubstrate Inhibitor	IC_50_ = 0.6 μM
Inauhzin	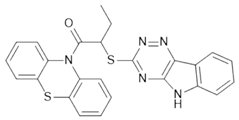	Non-nucleoside Inhibitor	IC_50_ = 23 μM EC_50_ = 12.96 μM (in Vero E6 Cells)
Z795161988	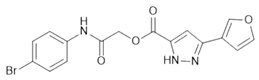	Non-nucleoside Inhibitor	IC50 = 2.2 μM
TDI-015051	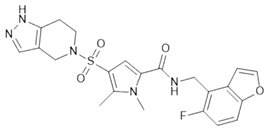	Non-nucleoside Inhibitor	IC50 ≤ 0.15 nM EC50 = 11.4 nM (in Huh-7.5 cell) EC50 = 64.7 nM (in ACE2-TMPRSS2 expressing A549 cell)

## Data Availability

No new data were created or analyzed in this study. Data sharing is not applicable to this article.
